# Progress for carbon dioxide geological storage in West Macedonia: A field and laboratory-based survey

**DOI:** 10.12688/openreseurope.15847.2

**Published:** 2023-08-09

**Authors:** Pavlos Tyrologou, Agnes Vamvaka, Nikolaos Koukouzas, Jorge Pedro, Marc Fleury, Julio Carneiro, Carlos Ribeiro, Dina Ghikas, Anna Mpatsi, João Pedro Barradas, Paula Faria, Fernanda De Mesquita Lobo Veloso

**Affiliations:** 1 Geo-RΕsources, EΝergy and environmental management, Centre for Research and Technology Hellas (CERTH), Egialias 52, Marousi, 151 25, Greece; 2 Institute of Earth Sciences and Department of Geosciences, University of Évora, Évora, 7000-671, Portugal; 3 IFP Energies nouvelles, Rueil-Malmaison, 92852, France; 4 Institute of Earth Sciences, Marine and Environmental Sciences Center, ARNET - Aquatic Research Network, Institute for Research and Advanced Training and Department of Geosciences, University of Évora, Évora, 7000-671, Portugal; 5 GeoBioTec and Department of Geosciences, University of Évora, Évora, 7000-671, Portugal; 6 Risk and Prevention Division Safety and Performance of Subsurface, Bureau de recherches géologiques et minières, Orléans, 45060, France

**Keywords:** MesoHellenic Basin, Carbon storage, geomechanics, petrophysics, climate change, porosity, permeability, uniaxial strength

## Abstract

**Background:** It is widely acknowledged that carbon dioxide (CO _2_), a greenhouse gas, is largely responsible for climatic changes that can lead to warming or cooling in various places. This disturbs natural processes, creating instability and fragility of natural and social ecosystems. To combat climate change, without compromising technology advancements and maintaining production costs at acceptable levels, carbon capture and storage (CCS) technologies can be deployed to advance a non-disruptive energy transition. Capturing CO _2 _from industrial processes such as thermoelectric power stations, refineries, and cement factories and storing it in geological mediums is becoming a mature technology. Part of the Mesohellenic Basin, situated in Greek territory, is proposed as a potential area for CO _2_ storage in saline aquifers. This follows work previously done in the StrategyCCUS project, funded by the EU. The work is progressing under the Pilot Strategy, funded by the EU.

**Methods:** The current investigation includes geomechanical and petrophysical methods to characterise sedimentary formations for their potential to hold CO _2_ underground.

**Results: **Samples were found to have both low porosity and permeability while the corresponding uniaxial strength for the Tsotyli formation was 22 MPa, for Eptechori 35 MPa and Pentalofo 74 MPa.

**Conclusions: **The samples investigated indicate the potential to act as cap-rocks due to low porosity and permeability, but fluid pressure within the rock should remain within specified limits; otherwise, the rock may easily fracture and result in CO _2_ leakage or/and deform to allow the flow of CO _2_. Further investigation is needed to identify reservoir rocks as well more sampling to allow for statistically significant results.

## Plain language summary

This publication presents the work of research institutes in their effort to address climate change via practical applications that foster job growth. It is well known that CO _2_ is a greenhouse gas released freely into the atmosphere, which is largely responsible for global warming. One solution is to use existing technology and capture CO _2_ from an industrial process such as thermoelectric power stations, refineries and cement factories. The captured CO _2_ will be stored forever, very deep into the ground, without risk of gas escaping to the atmosphere. Here, we try to see if available areas in West Macedonia in Greece offer the right underground conditions for safe CO _2_ storage. A team of researchers investigated a potential area close to Grevena and collected rock samples. All samples were found to be strong up to a limit with little pore space. The results show that the rocks are strong enough for safely trapping the CO _2_ and with very small pores to allow gas escape. To better understand the area, more work will be carried out to find rocks suitable for storing CO _2_. These will be deeper than the ones investigated and in an area that will not be affected by earthquakes.

## Introduction

Carbon Capture and Storage (CCS) technology plays a crucial role in achieving the goals of the Paris Agreement against climate change and the Intergovernmental Panel on Climate Change (IPCC) scenarios ^1^. The technology involves capturing carbon dioxide (CO _2_) from industrial activities and transportation pipelines and then storing it in secure geological reservoirs. Several capture technologies are available, including post-combustion capture, pre-combustion capture, oxy-fuel combustion, and chemical looping combustion ^2– 6^. After capturing CO _2_, it can be converted into various products and services such as fuels, chemicals, and building materials.

Geological storage provides the potential for permanently storing large quantities of CO _2_. There are several geological storage options available for mitigating the effects of climate change ^7– 11^, including deep saline aquifers, salt caverns, coal seams, abandoned coal mines, and depleted hydrocarbon fields ^3, 12– 17^. Enhanced oil and/or gas recovery (CO _2_-EOR and CO _2_-EGR) is another process that combines the extraction of crude oil and/or natural gas with simultaneous CO _2_ storage ^18– 20^. CO _2_-mineralizCO2 storage that involves the chemical reaction of several rock types with supercritical CO _2_, resulting in the formation of carbonate minerals and subsequent CO _2_ sequestration in the form of the formed carbonate minerals ^21– 23^.

The positive value applications of CO _2_ can also offset the cost of CCS technologies to store a tonne of carbon dioxide that range from $60 to $90 or €35 to €60 per tonne ^24– 26^ in the USA and Europe, respectively, where the geology is favourable. Prices can be higher where significant transportation is involved. There are some cases where cost can reach as high as €150 depending on the site requirements ^27^. The EU ETS price has been increasing since 2018, reaching a peak value in 27 February 2023 at 100.23 euros per tonne ^28^. Emerging capture technologies are even more promising, with a 40% cost reduction compared to current ones ^29, 30^.

There are several large-scale CCUS projects operating globally, with a CO _2_ capture capacity of 244 Mtpa ( https://www.gasworld.com/story/carbon-capture-and-storage-capacity-rises-to-244-mtpa/) ^31^. The Sleipner and Snovit projects in Norway are examples of successful CCS projects that have captured and stored 20 million tonnes of CO _2_ into deep offshore saline formations since 1996 ^32^. These projects provide valuable experience and lessons for CCS in Europe.

CCS technology can support the energy transition towards a low-carbon economy and achieve the European Green Deal's objectives ^33^; the EU response to the Paris treaty. The EU has established a framework for sustainable finance, including the EU Taxonomy, to facilitate the transition to a more sustainable economy. The EU Taxonomy provides a classification system for sustainable economic activities and aims to identify and promote investments in environmentally sustainable projects. It sets out criteria for economic activities that contribute to six environmental objectives, including climate change mitigation. CCS projects can qualify for the EU Taxonomy since they meet the technical screening criteria and other environmental, social, and governance criteria ^34^.

The EU supports the development of CCS technology through various funding mechanisms, such as the Innovation Fund, and the Horizon Europe programme ^35^. The Horizon 2020 provides financial support for innovative projects that reduce greenhouse gas emissions, including CCUS projects. The PilotSTRATEGY project is an Horizon2020 project that investigates geological CO _2_ storage sites in industrial regions of Southern and Eastern Europe to support the development of large-scale carbon capture and storage (CCS). It is the successor of the StrategyCCUS project, also funded by the Horizon 2020 programme and consequently builds upon the research funding of its predecessor. PilotSTRATEGY focuses on deep saline aquifers, porous rock formations filled with brine several kilometres below ground, which promise a large capacity for storing CO _2_ captured from industrial clusters ^31^. Detailed studies will be conducted on deep saline aquifers in the Paris Basin in France, the Lusitanian Basin in Portugal and the Ebro Basin in Spain. Knowledge enhancement for CO _2_ storage options are developed in Upper Silesia in Poland and the Mesohellenic trough in West Macedonia in Greece. The latter is the subject of this publication.

Previously, in STRATEGY CCUS a conservative geological modelling approach based on existing scientific literature attributed the Tiers1classification for the Mesohellenic Trough, which contains Pentalofos Formation with a CO _2_ capacity up to 1 Gt and Eptachori Formation with a storage capacity up to 0.85 Gt of CO _2_^31^. Further refinement of these initial estimations are being sought by characterising the storage complex to assess the site’s containment, injectivity, capacity, integrity, hydrodynamics, and monitorability to ensure safe and permanent storage of CO _2_.

### Geological setting

**Figure 1.**
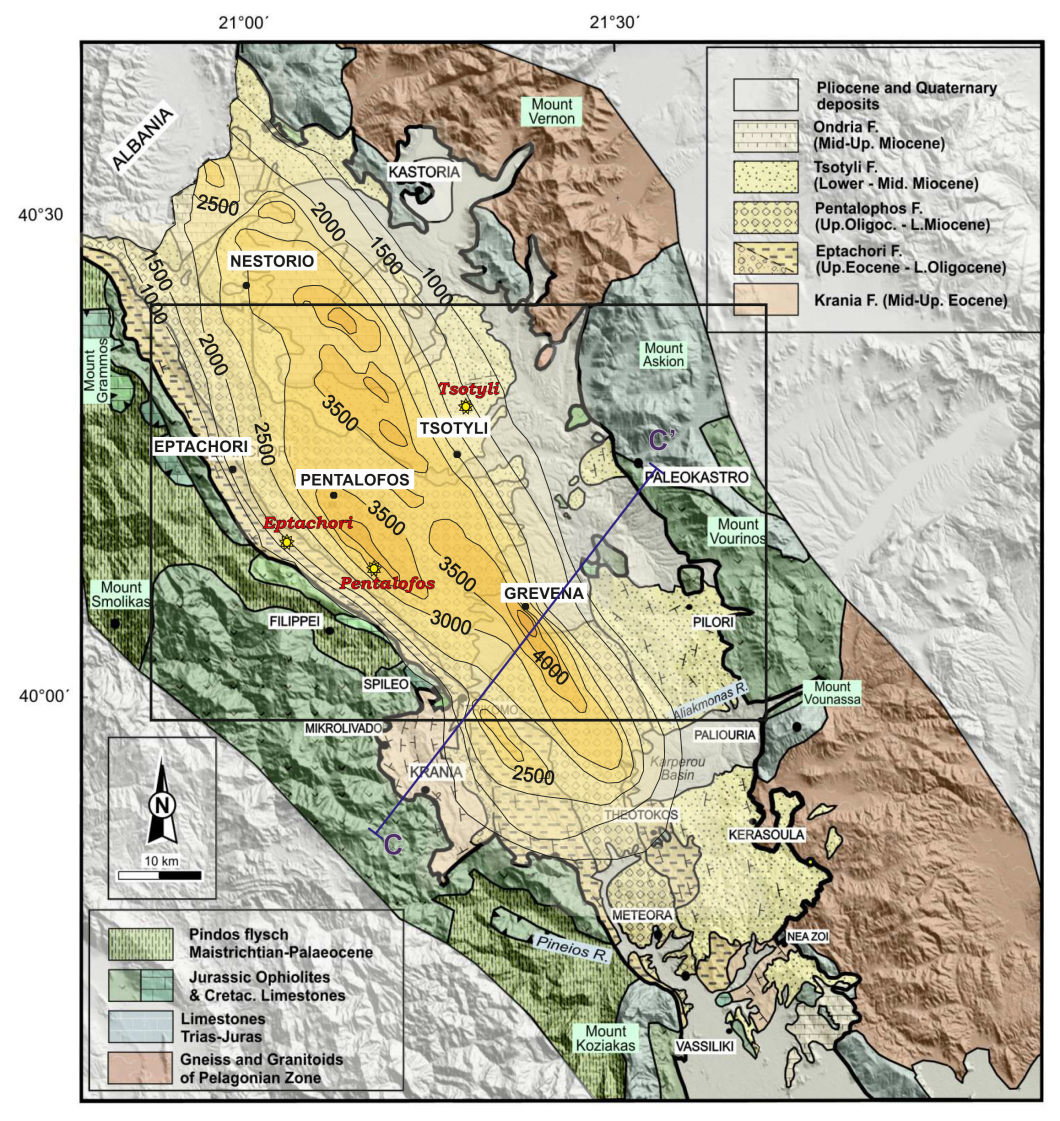
The Mesohellenic Basin: the main formations and isodepths of the basement rocks (modified and published with permissions from Vamvaka, 2009, 36). The framed area represents the selected sampling area, where the locations of the collected samples are illustrated as yellow star-points (i.e. three samples: Eptachori (EP), Pentalofos (PE) and Tsotyli (TS), respectively).

The Mesohellenic Basin (MHB) is a late-orogenic sedimentary basin formed during the Tertiary (Mid-Miocene) over the suture of the Apulian platform and the Pelagonian nappe ^36^ ( Figure 1), and is widely considered as the suture of the internal and external zones of the Hellenide orogenic belt ^37^. This basin developed along a NNW-SSE elongated axis, exceeding 200 km in length, while its width varies between 20 and 40 km. The basin extends from southern Albania to northwestern Greece, bordered by the main Greek orogenic range of Pindus in the West and the mountains Askion, Vourinos and Kamvounia in the East.

Tectonically, the entire area was affected by the last Alpine orogenic processes that outlasted the Tertiary, causing thrusting towards the west-southwest ^37^ and deformation of the Pindus Zone during the Middle-Late Eocene, which was emplaced over the External Hellenide zones. The Mesohellenic basin was formed during the latest stage of this orogenic event, on top of the westward overthrusted ophiolitic nappe ^36, 38^. The Pindus cordillera in the West encompasses the collision zone between the Apulian plate and Pelagonian continental nappe, the closure of the Tethys Ocean, and the westward emplacement of Tethyan ophiolite complex ^36, 39^. Rock types to the west of the MHB include ophiolitic and mélange units (Triassic-Jurassic), limestone (Cretaceous) and Pindus flysch (Maastrichtian-Palaeocene). In contrast, the eastern margin of the basin consists of the Pelagonian nappe rocks, including Pelagonian basement igneous intrusive/metamorphic rocks (Precambrian-Paleozoic) and rift-related rocks (Permian-Tr), as well as thrusted ophiolite, mélange and overlying Cretaceous limestones ^40^.

The MHB comprises five, mainly siliciclastic formations (i.e., Krania, Eptachori, Pentalofos, Tsotyli and Ondria Formations; Figure 1), which were deposited from the Late Eocene to the Middle Miocene. They show variations in thickness and facies across and along the basin axis ^36^. They include fan-delta conglomerates, alluvial fans, turbiditic sandstones and shales, deltaic and flood-plain sandstone and siltstones, and sandy shelf sediments ^41, 42^, which typically coarsen from North to South ^36^. Through progressive closure and shallowing of the seaway, the formations reflect an overall transition from the continental shelf to a terrestrial environment, with often abrupt facies changes and intercalations varying from turbiditic sandstones and shales to fan-delta conglomerates, deltaic and flood-plain sandstone and siltstones, and sandy shelf sediments ^41, 42^. The maximum vertical thickness of the sediment pile is 4–4.5 km near the Grevena area, while the cumulative thickness of the sediments is much greater.

**Figure 2.**
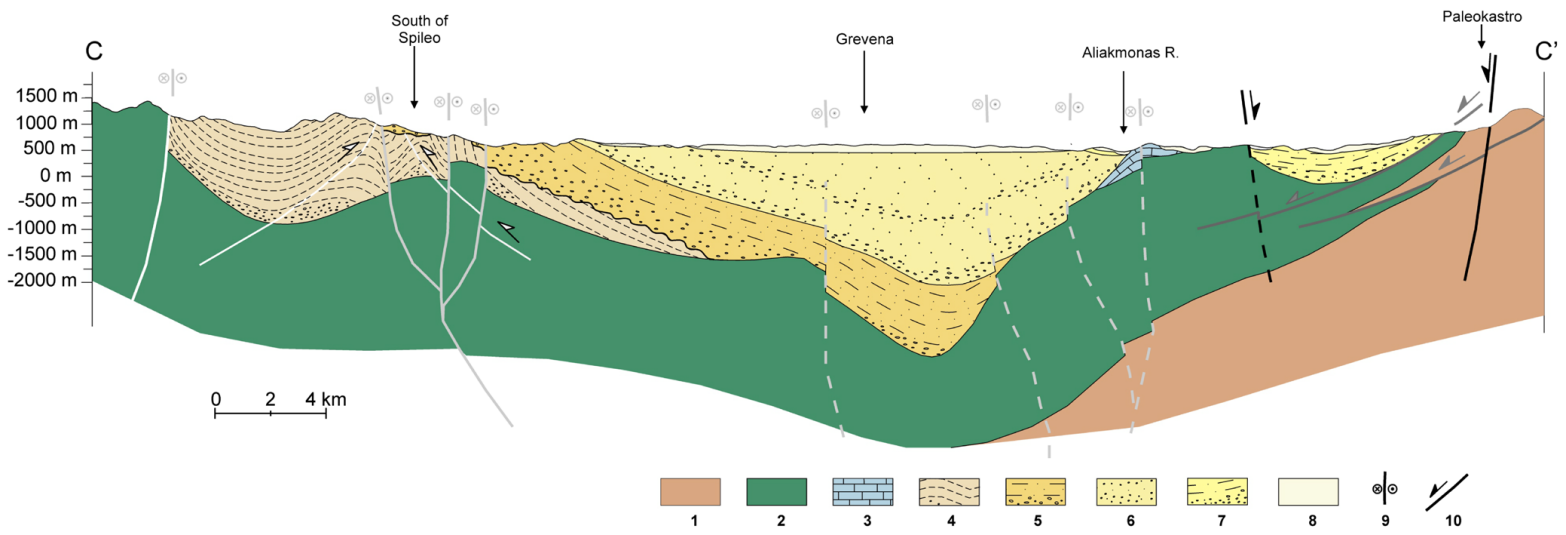
Cross-section from Krania in the West to Paleokastro in the East (cross-section reproduced with permission from Vamvaka, 2010), where 1: Pelagonian nappe, 2: Ophiolites, 3: Jurassic limestones, 4,5,6,7: Krania, Eptachori, Pentalofos, Tsotyli Fms, 8: Quaternary Deps, 9, 10: Strike-slip and dip-slip faults, respectively.

At the western boundary of the MHB, beds dip near-vertically, becoming more horizontal eastward and eventually dipping gently westward at the easternmost boundary of the basin. Thus the basin forms an asymmetrical syncline, as confirmed by field observations ^36^ and seismic profile interpretations ^41^. In the southern part of the basin, the MHB is subdivided into two basins by the Theotokos-Theopetra Structure ( Figure 2), which is a horst or faulted anticline trending approximately parallel to the NNW-SSE strike of the MHB and exposing basement ophiolitic and limestone units ^36, 43, 44^. It forms a structural high, with depocenters to the west and east of it.

The inclination of the bedding is related both to the primary deposition gradient and tectonic activity. Except for the Theotokos-Theopetra Structure in the South, the western basin boundary is recognized as a great fault of NNW-SSE orientation (Vamvaka, 2010). NNW-SSE faults and WSW-ENE have also been recorded within the basin, cutting mainly the Eptachori and Pentalofos strata and thus associated with the late Eocene-Oligocene period of their deposition ^36^ (Vamvaka, 2010). Extensional faults from the beginning of the Miocene are also documented along the eastern basin boundary and within the basin, with varying directions from NW-SE to ENE-WSE, depending on the changing orientation of the main extensional stress axis (σ1) from NE-SW to the N-S ^36, 38, 45^.

Both the main NW-SE and the NE-SE to ENE-WSW structural directions are followed by several rivers and their tributaries (i.e., Aliakmonas, Ionas and Pinios rivers), and thus related to pre-existing fracture zones, some possibly reactivated as normal faults under the younger extensional regime ^36^. The present *ca* N-S extension is considered capable of generating significant seismic activity, as shown by recent examples i.e., earthquake activity in Grevena-Kozani areas in 1995, 2015 and 2021 ^46^.

## Methods

This section deals with the sampling from the appropriate geological formations of interest and the characterisation of the samples collected using geomechanical and petrophysical methods. Where appropriate, a brief theoretical background is provided.

### Sampling campaign

The selection of the sampling area was performed, taking into account the characteristics and limitations of the study. The basin area for CO _2_ storage must be of significant size to ensure a meaningful storage volume through cost-effectiveness. Such basic parameters are ^47^: (i) great thickness of clastic deposits, since the minimum depth for CO _2_ injection is 800 meters, (ii) an impermeable caprock to avoid any leaking, (iii) an appropriate porosity at depth so that the lower sedimentary layers can host a considerable volume of injected CO _2_, (iv) suitable hydrological conditions to avoid any cross contamination of the aquifers, and (v) a lack of deep active fractures or major fault zones that may be reactivated under the present stress regime.

Taking into account the available published data ^36, 41– 44, 46, 48– 50^ and in situ observations, a suitable candidate area for sample representativeness was considered to be across the central–northern part of the MHB, where the basin has its greatest development both in width and depth ( Figure 1). Three main MHB formations occur in this area: Eptachori, Pentalofos and Tsotyli. The oldest, Krania Fm, and the youngest, Ondria Fm were only deposited or preserved in places and therefore do not compose a standard sedimentary bed.

The total maximum vertical thickness of the deposits is estimated to be ≥ 4,000 meters in places, based on the interpretation of seismic profiles. In contrast, the accumulative thickness of the deposits exceeds 6–7 km ^41^, Figure 2. Published data regarding the porosity of the lower Pentalofos and Eptachori strata, which could serve as CO _2_ reservoir, provide estimated porosity values between 7 and 25% ^31, 48, 49, 51^. Although there is no analysis or estimations for the porosity of the overlying Tsotyli Formation strata, most beds are resistant and minimally deformed and hence could be considered as the caprock to the East. For the western areas not covered by the Tsotyli strata, the higher layers of Pentalofos and Eptachori Formations could potentially serve as cap-rock themselves because they consist of alternating layers with alternating different characteristics, some very fine-grained and thus of no or extremely low porosity, which predicts conditions of very low permeability ^52, 53^. The clearly permeable formations are the shallow Quaternary alluvial deposits, which have the older molassic formations as a bottom impermeable barrier. The depth of the groundwater level ranges from close to the surface to up to 50 meters ^54^.

Regarding the presence of deep fault structures, there is not enough data that could be considered at this point. Since faulting is recorded as a basic factor during the basin formation, there are certainly pre-existing fault zones, but those are mainly traced along the basin boundaries ^36, 43, 44^. There is no certain proof of fault structures all along the longitudinal centre of the basin, like the ones noted at Theotokos-Vassiliki area in the South (Vamvaka, 2010), which renders the selected sampling area more suitable for CO _2_ storage. However, faults of ENE-WSW to NE-SW direction are also reported within the basin area to have acted simultaneously with the main marginal NNW-SSE faults of MHB, but also related to more recent activity ^46^.

**Figure 3.**
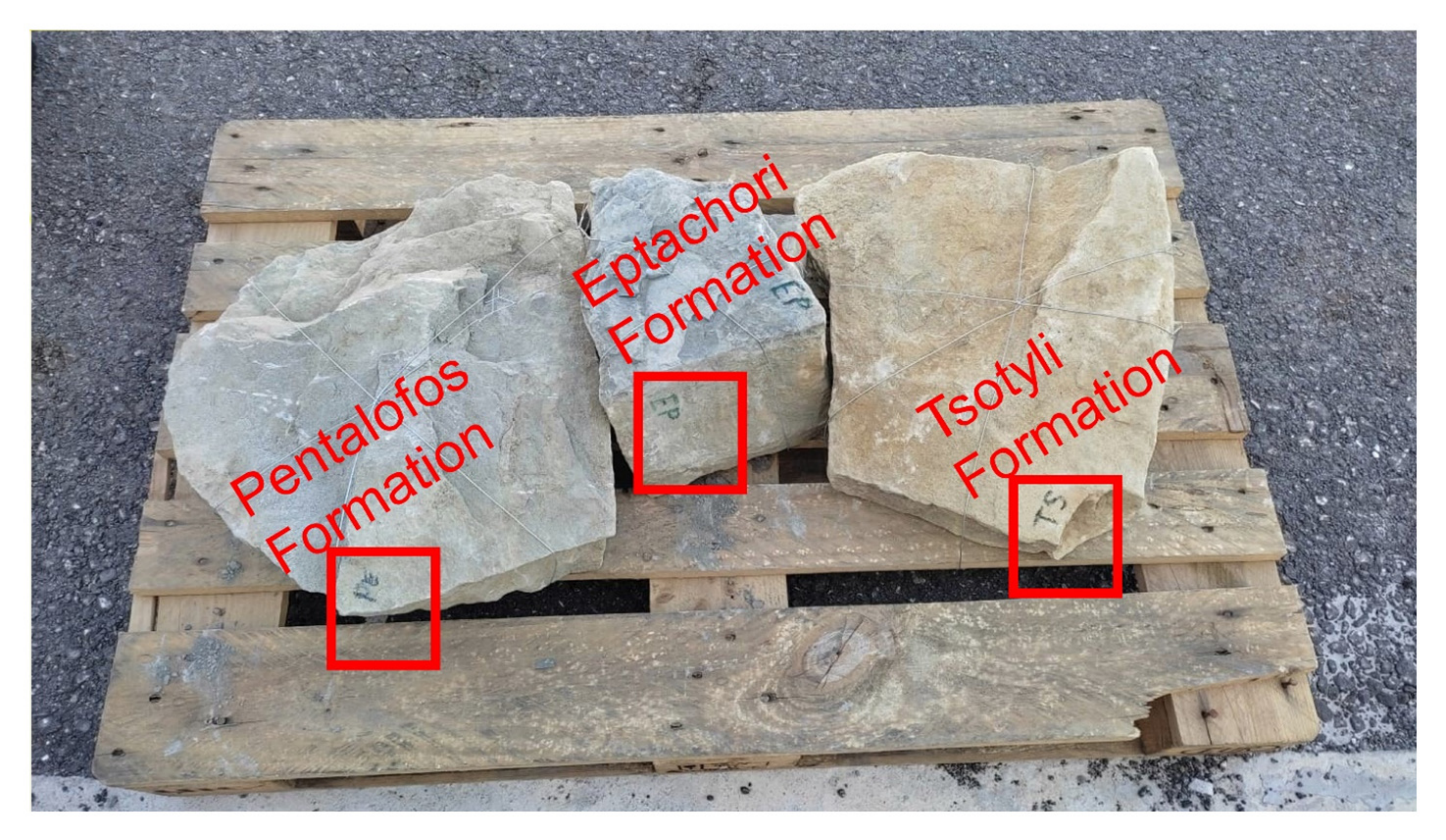
Bulk samples collected during the walk over survey and analysed in France by French Institute of Petroleum (IFP) Energies nouvelles – Earth Sciences and Environmental Technologies and in Portugal by the Departamento de Geociências Universidade de Évora for petrophysical and geomechanically laboratory investigation respectively.

From December 2021 to May 2022 several walk-over surveys were conducted to gather an initial data set. During these surveys, samples from the Tsotyli, Pentalofos and Eptachori formations were collected and subsequently sent to various laboratories for petrophysical and geomechanical investigation ( Figure 3).

The chosen samples were selected from intermediate parts of each formation and locations to represent each formation overall (i.e., in terms of composition, considering the whole of their development across the central part of the basin). The locations of the samples are displayed on the map in Figure 1 and their exact co-ordinates are provided in section 3.2.

### Field samples description

The field sampling description has been conducted according to BS 5930:2015+A1:2020 ^55^. Stratigraphically from the younger to the older, the samples are described below.

*Tsotyli Formation (Lower-Middle Miocene), WGS84 sample coordinates Lat : 40.3075, Long : 21.3354.*

Alternation between units of varying grain size and strength: 1. 0.5-1.5m-thick beds of medium weak to very strong, partially weathered, grey CONGLOMERATE. Clasts are poorly sorted (0.5-10+mm with occasional larger clasts), sub-angular to sub-rounded, predominantly limestone with igneous/metamorphic clasts and fossil corals, grain-supported with clastic matrix. No interior bedding or structures. 2. 10cm-1m-thick beds of medium weak to very strong, partially weathered, grey greywacke. Grains are fine, angular, limestone-quartz-micas-various mafics.

*Pentalofos Formation (Upper Oligocene to Lower Miocene), WGS84 sample coordinates Lat : 40.1332, WGS84 Long : 21.1997.*

Slightly weak to medium strong beds of partially weathered, grey SANDSTONE. Grains are fine, crystalline, most of them are indistinguishable from the matrix. Many mica and mafic grains. Sample effervesces in acid—either a calcareous matrix or limestone grains (could not be determined macroscopically). Some weak interior bedding. Occasional trace fossils (burrow casts). Iron oxide staining.

*Eptachori Formation (Uppermost Eocene – Lower Oligocene), WGS84 sample coordinates Lat : 40.1535, Long : 21.0824.*

Very strong, thickly bedded (20-30cm), partially weathered, medium grey-tan, fine GREYWACKE. Joint fractures spaced 40-80cm apart, perpendicular to bedding. Trace fossils (invertebrate burrows) on bedding surfaces. Partially carbonised wood and leaf fragments. Water discolouration (Liesegang) penetrates 8-10cm into the bedding.

The data from the samples collected during the survey conducted for the purposes of the current work described in this publication, was uploaded to the System for Earth Sample Registration ( SESAR) platform. This enables the data to be Findable, Accessible, Interoperable, and Reusable (FAIR) via unique sample identifiers. The data from the collected samples are available in the SESAR platform as follows:

1. Tsotyli formation: https://app.geosamples.org/sample/igsn/IE5770001
2. Pentalofos formation: https://app.geosamples.org/sample/igsn/IE5770002
3. Eptachori formation: https://app.geosamples.org/sample/igsn/IE5770003

### Geomechanical laboratory investigation

Geomechanical characterisation of the Tsotyli (TS), Pentalofios (PE) and Eptachori (EP) formations was conducted through standard laboratory tests performed at the Geosciences Department Laboratory and at the Laboratory of Mechanical Tests (LEM) of the University of Évora. Representative samples were collected (see previous section) at outcrops and tested for the required parameters using:

1. P-wave velocity (Vp)
2. Point Load Test and
3. Schmidt Hammer methods

Dynamic Elasticity Modulus (Ed) and material density can be estimated from the p-wave velocity (Vp). The geomechanical methods implemented are briefly discussed below.

***p-wave velocity determination*.** For the P-wave propagation velocity (Vp) a PUNDICT PL 200 with 54kHz transducers apparatus was used following the British Standard BS 1881 Part 203 ^56^. Two transducers were placed at the opposite sides of a test specimen of length L. One of the transducers emits sound waves that propagate through the specimen and are received by the other transducer. Vp is the ratio between L and the time lapse between the emission and the receiving of the sound pulse. Dynamic Elasticity Module (Ed) can be determined using Equation 1 ^56^:


$$E_{d}=\rho V^{2}\times \frac{\left(1+v)\times (1-2v\right)}{\left(1-v\right)}\;\text{Equation 1}$$

Where *E _d_* is the dynamic elastic modulus (Pa), ν is the Poisson’s ratio, ρ the density (kg/m ^3^) and V is the pulse velocity (m/s).

Poisson ratio was also calculated using Equation 2 ^57^, Vp (m/s) and Vs (m/s) being the propagation velocities of P-waves and S-waves.


ρ=(VpVs)2−22[(VpVs)−1]Equation 2

For each sample, 7 cubes were cut at 5 × 5 × 5 cm. The results of the measurements were subsequently averaged.

***Point Load Test*.** Point Load Test was done following the standard ASTM D 5731-95 of ASTM International ^58^. The equipment consisted of a loading system produced by ELE with the measurement of the applied load (P) consisting by two rigs that can operate at 5.6 kN and 56 kN. Conical tips were applied to opposite sides of the sample.

Prismatic samples of a square base with 5 cm edge and 10 cm height were used (due to the impossibility to produce cylindrical samples). This geometry is equivalent to that provided for the test on a cylindrical sample; hence the result obtained from the tests does not need to have any correction applied. The resulting *I _s_* value is equal to the *I _50_* value.

*I _s_* value can be determined from the Equation 3 where *P* is the failure load (kN) and *D _e_* ( *m*) is the equivalent core diameter.


$$I_{s}=\frac{P}{D_{e}^{2}}\;\text{Equation 3}$$

D _e_^2^ equals D ^2^ (the diameter of the core) for diametral tests or 4A/π for axial, block and lump tests (ASTM D 5731-95) ^58^.

Seven prism per sample were tested and the average value of the observations was calculated. From the values of *I _50_*, tensile strength, uniaxial compressive strength and elasticity modulus were estimated using the empirical relations of the literature.

***Schmidt hammer*.** The Schmidt hammer is a device that measures the contact resistance of a material. Initially designed to test concrete, it is also used to test the strength of rocks. The equipment has a plunger that transmits the impulse, a system of springs and a graduated scale that allows measuring the resistance to impact (rebound). The hammer is armed; the plunger is placed against the specimen to be tested, the system is triggered by releasing the plunger, and the rebound value marked on the scale is recorded.

The equipment has no geometrical constraints, allowing the resistance to be determined on any sample surface without prior treatment. The test is performed several times to determine an average value. Using the obtained values and knowing the density of the tested sample, the uniaxial compressive strength and elasticity modulus can be determined using empirical relations.

### Petrophysical laboratory investigation

Petrophysical information such as porosity, pore size distribution, bound and movable water and permeability can be obtained using nuclear magnetic resonance (NMR) methods. An NMR measures only pore fluids and NMR porosity is matrix independent ^59, 60^.

The petrophysical investigation was carried out in the IFP Energies Nouvelles in France laboratories utilising Nuclear Magnetic Resonance techniques. The instrument is the Rock Core Analyzer from Magritek. A Carr-Purcell-Meiboom-Gill (CPMG) sequence was used to obtain transverse relaxation times T _2_ from the CPMG envelope. An interecho spacing of 0.1ms and up to 25 000 echoes were used in all the measurements. The number of scans is such as to reach a signal to noise ratio of 100. The T _2_ (ms) relaxation time distribution is a proxy of the pore size $\frac{v}{s}$ as described by Equation 4 valid when the bulk relaxation time of the saturating fluid is much larger than the measured relaxation T _2_. The s is the pore surface and v is the fluid volume, p _2_ is the T _2_ surface relaxivity (T _2_ relaxing strength of the grain surfaces). 


$$\frac{\text{v}}{\text{s}}={\text{ρ}}_{2}{\text{T}}_{2}\;\text{Equation 4}$$

Together with porosity, T _2_ can be used to evaluate permeability. In addition to NMR, the following petrophysical measurements were performed ^59^:

● Permeability measured with brine (NaCl 20g/l),
● formation factor FF measured during permeability estimation from which a
● single point cementation exponent m such as FF=Φ ^-m^ is calculated.

The flooding experimental device used has a range of measurable permeabilities starting at 0.01mD. Below this limit, permeability measurements are very time consuming using standard protocols. In the present study, samples were not transferred to a more specific device able to determine very low permeabilities (down to nD) and gas entry pressures. Hence, when the lower limit is reached, the value <0.01 mD is indicated. Five cylindrical samples with diameter e.q. 40 mm and height from 60 up to 80 mm were cored out of the bulk samples received and prepared accordingly for NMR scan and permeability test.

A very useful information that can be obtained from NMR is the Clay-bound- water (CBW), the amount of water located in clays (i.e., small or very small pores including interlayer water). It is obtained with a standard cut-off of 30 ms, calculated from a T _2_ distribution measured at Sw e.q. 100% with brine 20 g/l NaCl.

**Figure 4.**
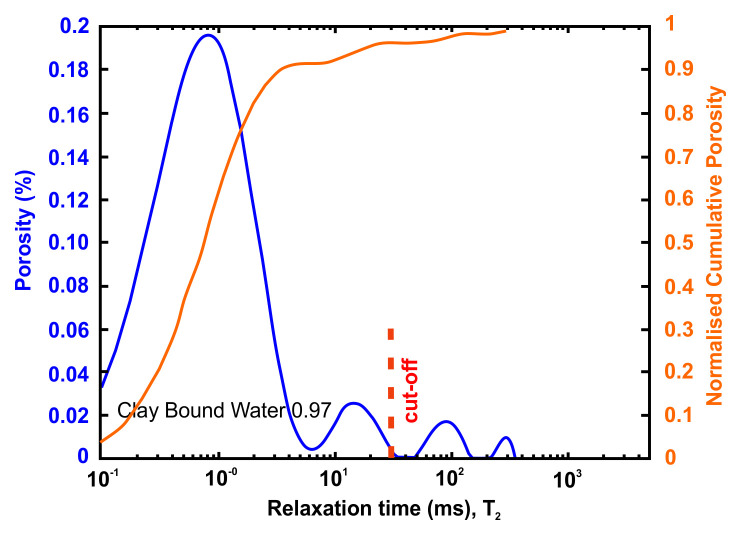
Example of a nuclear magnetic resonance (NMR) result and interpretation. The area under the curve is the total porosity (in %). A standard cut-off value at 30 ms defines the amount of water located in clays. This cut-off can however vary depending on the value of surface relaxivity r2 (i.e. the type of clays).

Figure 4 below presents typical result from one of the samples after an NMR run. In this example, about 97% of the porosity is located in clays. For CO _2_ application, it means that only 3% at best of the porosity can be used for storing CO _2_ since the pressure necessary to invade the small pores in the clays is much too large in practice.

## Results

The convention used for the sample identification is as follows: a) TS corresponds to Tsotyli formation samples, b) EP corresponds to Eptachori samples, c) PE corresponds to Pentalofos formation samples. Please see *Underlying data* ^61, 62^ and *Extended data* ^61^ sections at the end of the manuscript for access to the full data associated with the results.

### Geomechanical data results

The petrophysical laboratory investigation for the Mesohellenic basin samples was conducted by the Institute of Earth Sciences and Department of Geosciences of University of Évora. The raw data can be retrieved from the Zenodo repository ^62^.

***Dynamic Elasticity modulus*.** For each sample, seven cubes were prepared with dimensions 5cm × 5cm × 5cm and subsequently were tested along the 3 possible directions. The results are presented in Table 1.

**Table 1.** Dynamic Elasticity modulus (Ed) obtained from P-wave propagation speed (Vp).

Sample	Average V. (GPa)	Standard deviation
**TS**	2.5	0.1
**EP**	26	1.1
**PE**	38	2.3
Correlation C1 – Ed		

***Point Load Strength Index Test*.** Geomechanical parameters such as Tensile Strength (BTS), Uniaxial Compressive Strength (UCS) and Elasticity Modulus (E) can be estimated from the point load test using correlation equations found in the literature.

The test was done in seven prisms with a square base of 5cm × 5cm and 10 cm in height. With this geometry, there is no need to introduce a correction factor whereby ls =ls(50). The standard used for the point load determination was ASTM D 5731-95 ^58^.

The determined values of Point Load Strength Index for the studied sampled and the estimated values of BTS ( Table 2), UCS ( Table 3) and E ( Table 4) are presented below.

**Table 2.** Average Tensile Strength (BTS) obtained from point load test. Stdev stands for standard deviation.

Sample	V. (MPa)	Stdev	V. (MPa)	Stdev	V. (MPa)	Stdev	V. (MPa)	Stdev
Correlation	C1 - BTS		C2 - BTS		C3 - BTS		C4 - BTS	
**TS**	1.1	0.1	1.5	0.1	0.85	0.06	1.1	0.07
**EP**	1.6	0.18	2.3	0.3	1.3	0.1	1.6	0.2
**PE**	2.8	0.2	4.3	0.4	2.4	0.28	3.1	0.29

**Table 3.** Uniaxial Compressive Strength (UCS) obtained via Point load test.

Sample	Average V. (GPa)	Standard deviation
**TS**	22	1.7
**EP**	35	5.0
**PE**	74	8.0
Correlation C5 - UCS		

**Table 4.** Elasticity Modulus (E) obtained via the point load test.

Sample	Average V. (GPa)	Standard deviation
**TS**	14	0.8
**EP**	20	2.3
**PE**	36	3.2
Correlation C3 - E		

***Schmidt Hammer Test*.** Schmidt Hammer test allows the determination of the material’s resistance to the impact of the hammer shoot (rebound resistance). In conjunction with the sample density, this parameter can be used to estimate the Uniaxial Compressive Strength (UCS) by using the published numerical correlation between the rebound resistance and UCS. Results are presented in Table 5 and Table 6.

**Table 5.** Uniaxial Compressive Strength (UCS) obtained via Schmidt Hammer, direct results.

Sample	Average V. (MPa)	Standard deviation
**TS**	31	4.5
**EP**	35	3.5
**PE**	56	7.0

**Table 6.** Uniaxial Compressive Strength (UCS) obtained via Schmidt Hammer, correlated results.

Sample	V. (MPa)	Stdev	V. (MPa)	Stdev	V. (MPa)	Stdev	V. (MPa)	Stdev
Correlation	C1 - UTS		C2 - UTS		C3 - UTS		C4 - UTS	
**TS**	158	6.0	43	5.0	51	5.9	28	12.0
**EP**	61	19.0	60	5.0	71	5.6	71	11.5
**PE**	188	56.0	79	5.8	94	6.8	117	14.0

The Schmidt-Hammer test also can be used to calculate the Elasticity Modulus (E), using numerical approaches from published papers. Results are presented in Table 7.

**Table 7.** Elasticity Modulus (E) via Schmidt Hammer.

Sample	Average V. (GPa)	Stdev	Average V. (GPa)	Stdev
Correlation	C1 - E		C2 - E	
**TS**	95	11.0	10	3.4
**EP**	86	6.7	30	7.4
**PE**	126	9.2	72	17.2
Correlation C1 - E				

***Petrophysical data results*.** The petrophysical laboratory investigation for the Mesohellenic basin samples was conducted by the IFPEN. The permeability was measured with brine (NaCl 20g/l). All permeabilities were too low to be measured with the device used. An upper limit is given instead. The Formation factor FF was measured during permeability estimation while a single point cementation exponent m such as FF=Φ ^-m^ was adopted. The results of the petrophysical analysis from this current study are presented below. The raw data can be retrieved from the Zenodo repository ^61^.

**Figure 5.**
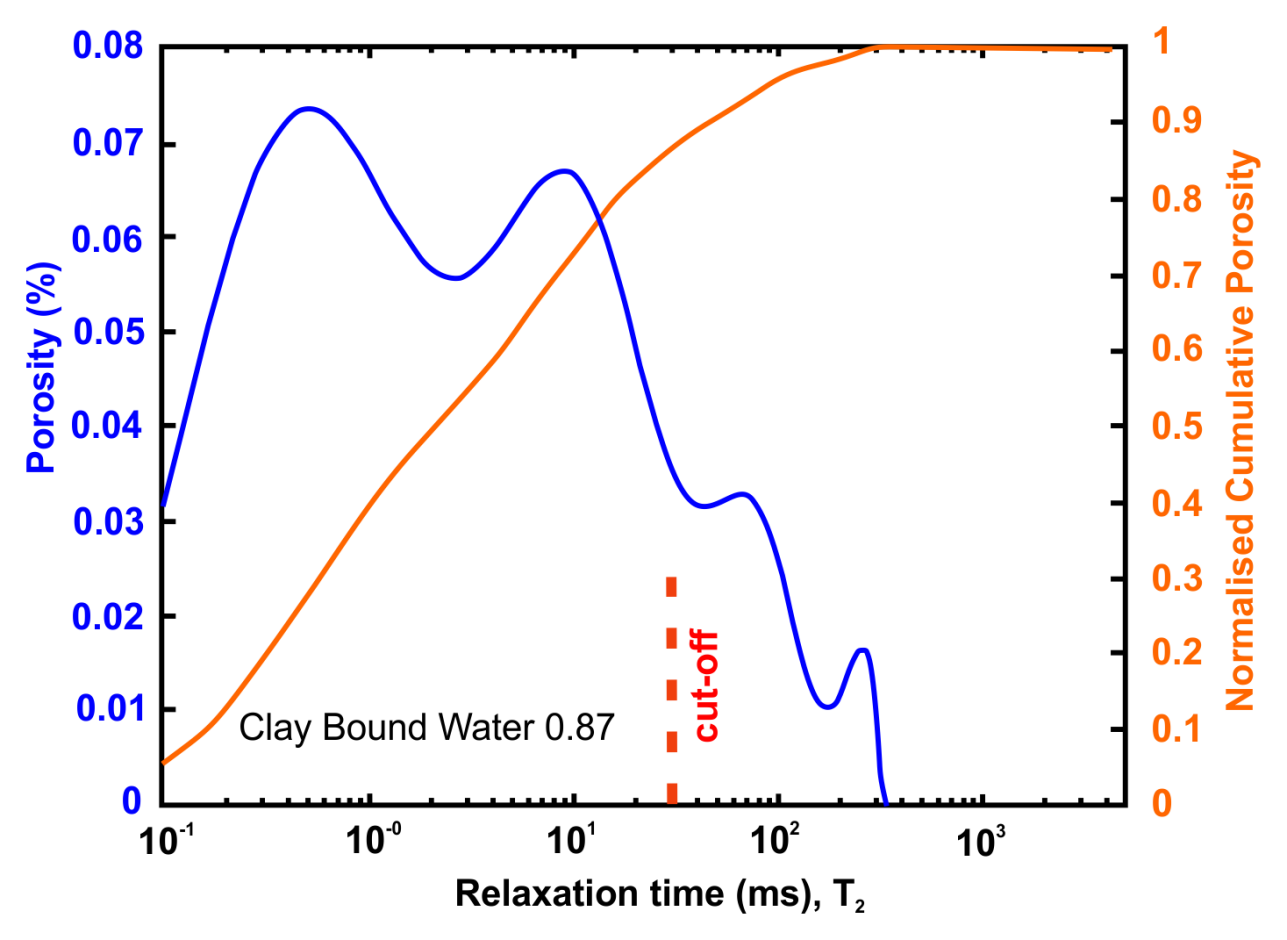
Porosity and cumulative porosity values for sample TSOT-1-3 (Tsotyli formation) from current study, time-cut off at 30 ms.

***Petrophysical results for Tsotyli formation*.** Table 8 tabulates the results from Figure 5 and both present the petrophysical results for the Tsotyli Formation (Lower Miocene, estimated thickness 1700 m).

**Table 8.** Petrophysical laboratory results for sample TSO 1-3 collected from the Tsotyli formation.

Petrophysical Properties	Values	Sample code: TSO-1-3 WGS84 Lat : 40.3075 WGS84 Long : 21.3354
**Porosity (%)**	6.0	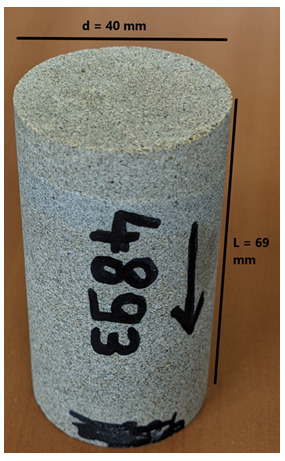
**Water Permeability (mD)**	<0.01	
**Formation Factor/m**	273/1.99	
**Clay bound water (fraction)**	0.87	

**Figure 6.**
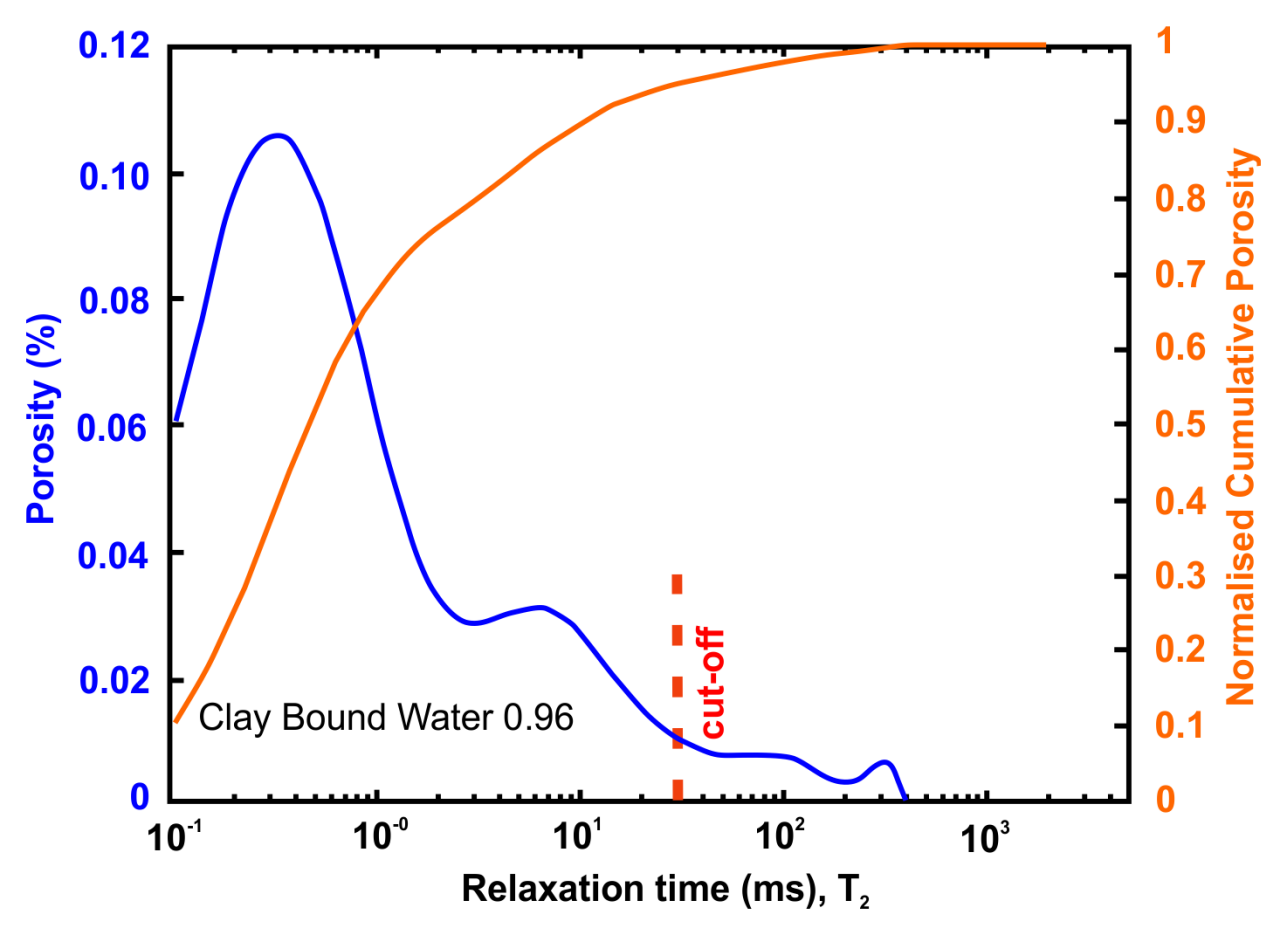
Porosity and cumulative porosity values for sample PENT-3-1 (Pentalofos formation) from current study, time-cut off at 30 ms.

***Petrophysical results for Pentalofos formation*.** For the Pentalofos formation, three samples were cored from the bulk sample and extracted for petrophysical investigation. Since and the three samples come from the same batch, they share the same geographical coordinates. Table 9 tabulates the results from Figure 6 and both present the petrophysical results for the sample Pent 3-1 from the Pentalofos Formation (Upper Oligocene - Lower Miocene, estimated thickness 2500 m).

**Table 9.** Petrophysical laboratory results for sample PENT-3-1 collected from the Pentalofos formation.

Petrophysical Properties	Values	Sample code: PENT 3-1 WGS84 Lat : 40.1332 WGS84 Long : 21.1997
**Porosity (%)**	5.0	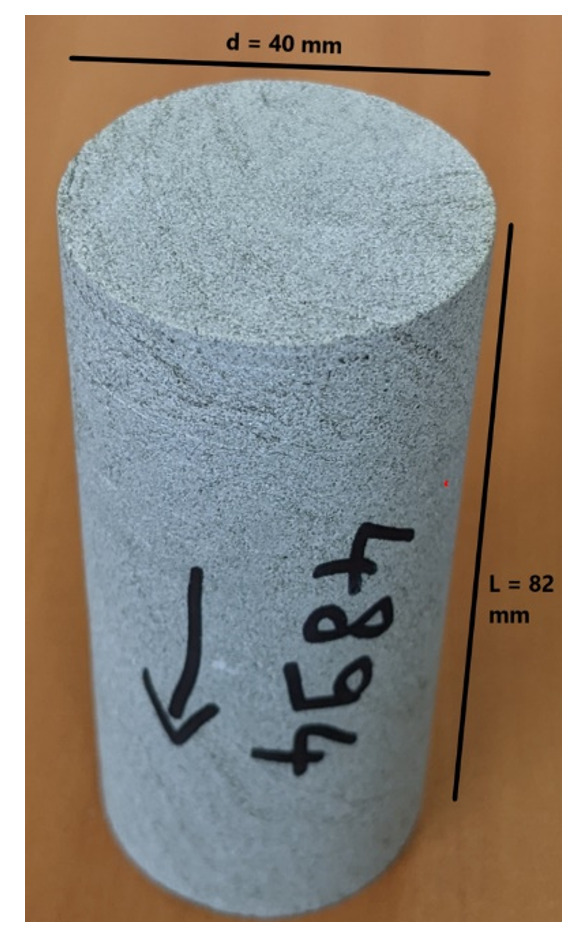
**Water Permeability (mD)**	<0.01	
**Formation Factor/m**	112/1.58	
**Clay bound water (fraction)**	0.96	

**Figure 7.**
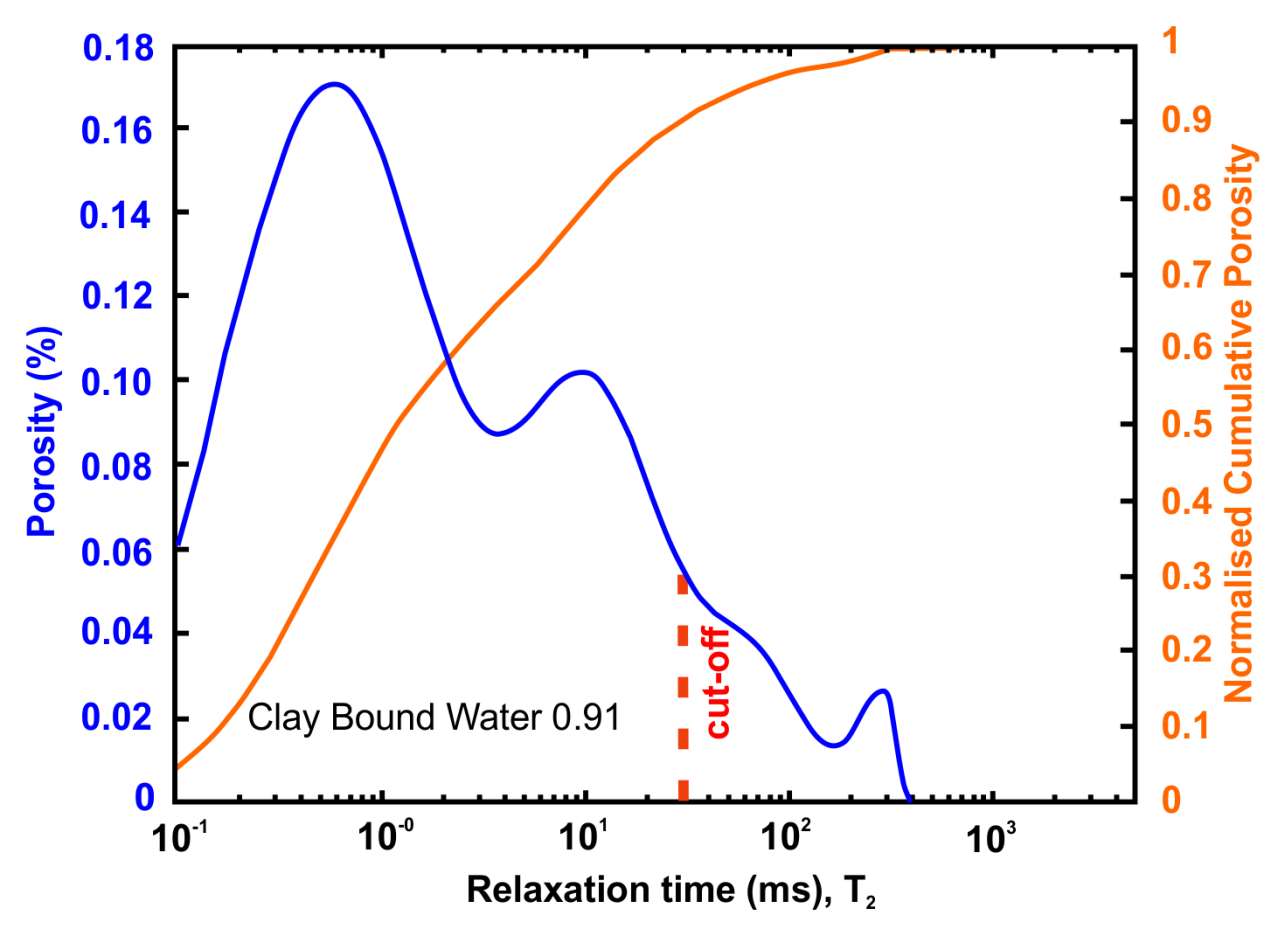
Porosity and cumulative porosity values for sample PENT-3-2 (Pentalofos formation) from current study, time-cut off at 30 ms.

Table 10 tabulates the results from Figure 7 and both present the petrophysical results for the sample Pent 3-2 from the Pentalofos Formation.

**Table 10.** Petrophysical laboratory results for sample PENT-3-2 collected from the Pentalofos formation.

Petrophysical Properties	Values	Sample code: PENT 3-2 WGS84 Lat : 40.1332 WGS84 Long : 21.1997
**Porosity (%)**	**10.8**	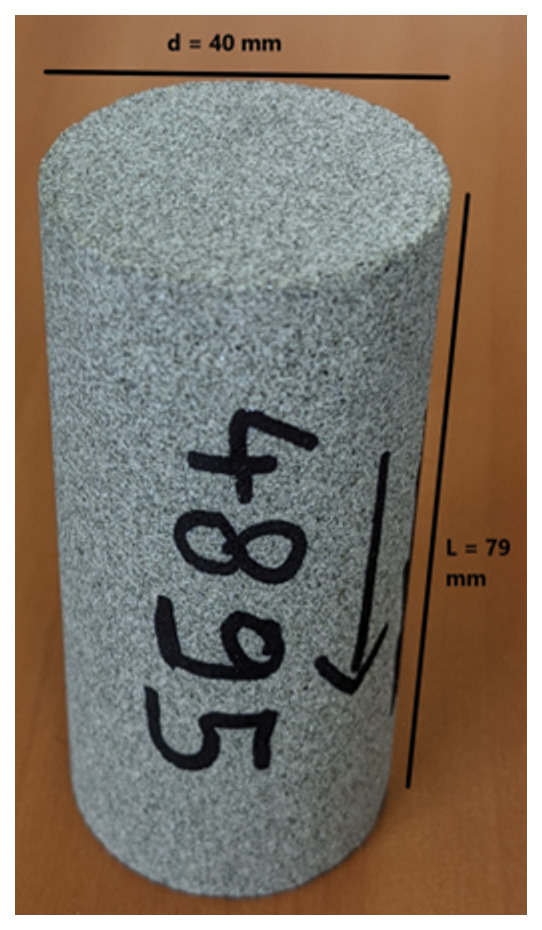
**Water Permeability (mD)**	<0.01	
**Formation Factor/m**	46/1.72	
**Clay bound water (fraction)**	0.91	

**Figure 8.**
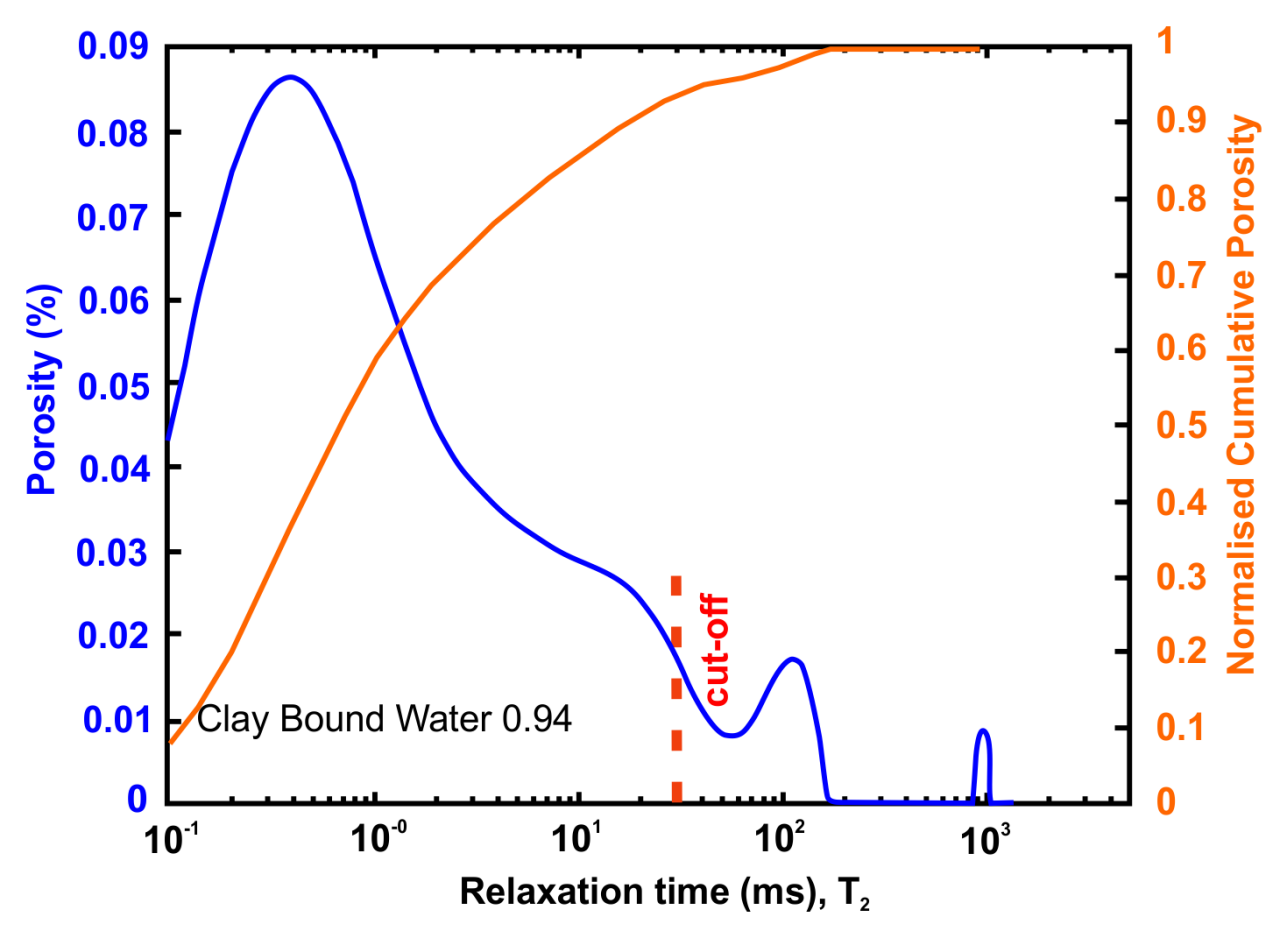
Porosity and cumulative porosity values for sample PENT-3-3 (Pentalofos formation) from current study, time-cut off at 30 ms.

Table 11 tabulates the results from Figure 8 and both present the petrophysical results for the sample Pent 3-3 from the Pentalofos Formation.

**Table 11.** Petrophysical laboratory results for sample PENT-3-3 collected from the Pentalofos formation.

Petrophysical Properties	Values	Sample code: PENT 3-3 WGS84 Lat : 40.1332 WGS84 Long : 21.1997
**Porosity (%)**	4.9	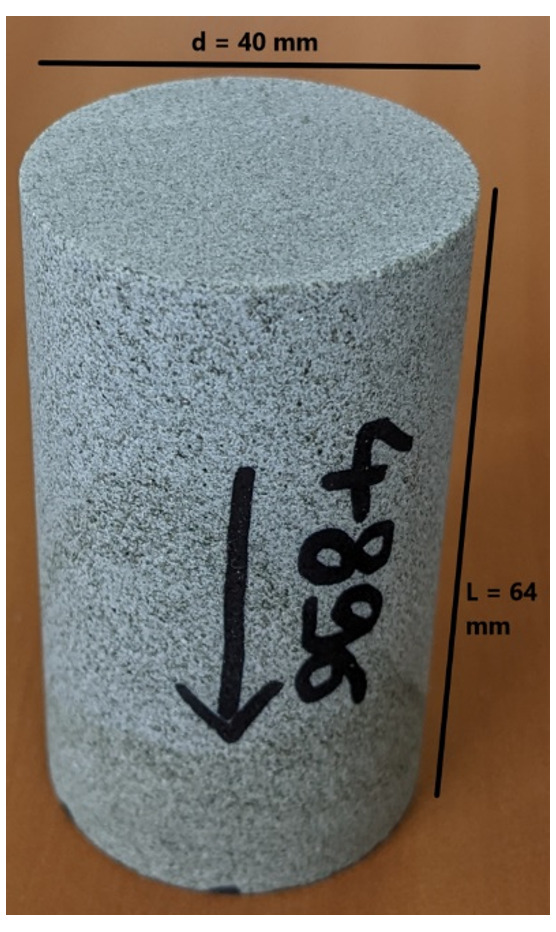
**Water Permeability (mD)**	<0.01	
**Formation Factor/m**	157/1.68	
**Clay bound water (fraction)**	0.94	

**Figure 9.**
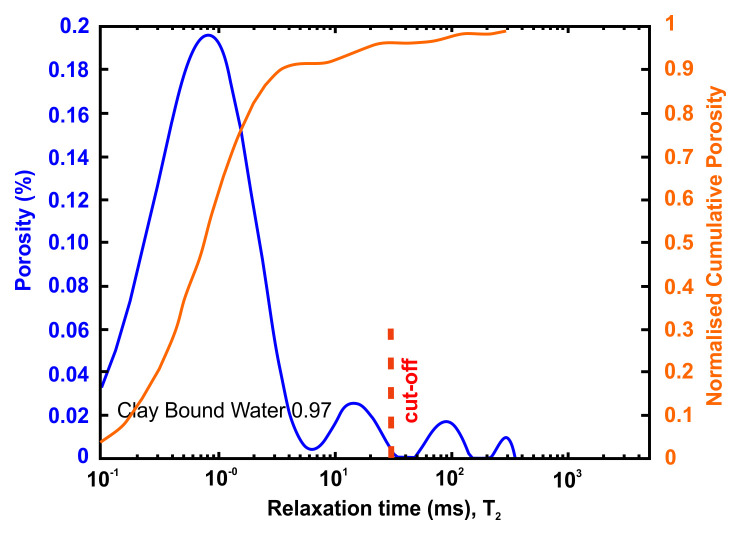
Porosity and cumulative porosity values for sample EPT-2-3 (Eptachori formation) from current study, time-cut off at 30 ms.

***Petrophysical results for Eptachori formation*.** For the Eptachori formation one sample was cored from the bulk sample and extracted for petrophysical investigation. Table 12 tabulates the results from Figure 9 and both present the petrophysical results for the sample EPT 2-3 from the Eptachori Formation (Lower - Upper Oligocene), estimated thickness 1500 m).

**Table 12.** Petrophysical laboratory results for samples collected from the Eptachori formation.

Petrophysical Properties	Values	Sample code: EPT-2-3. WGS84 Lat : 40.1535, WGS84 Long : 21.0824
**Porosity (%)**	7.4	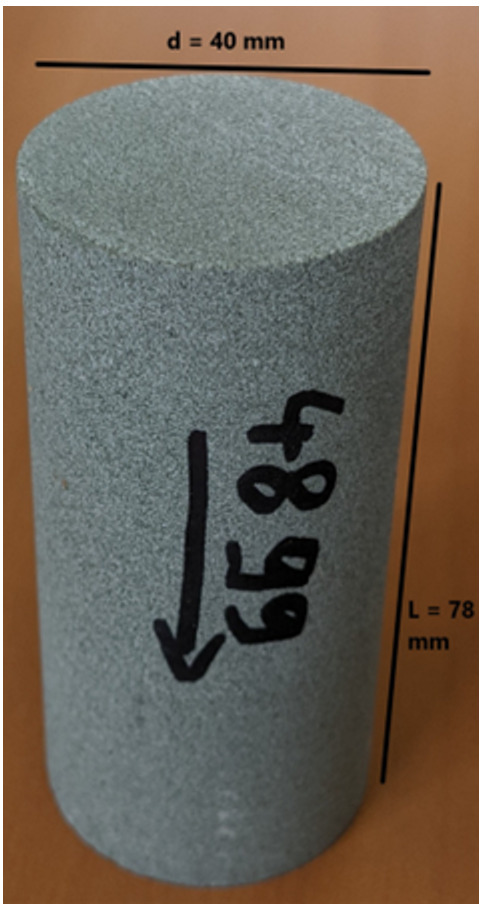
**Water Permeability (mD)**	<0.01	
**Formation Factor/m**	123/1.46	
**Clay bound water (fraction)**	0.97	

## Discussion

The samples collected during the walk-over survey are indicative and represent the first attempt to understand the potential conditions in the area. However, they have been collected randomly and are neither based on a statistical sampling framework nor a focused survey. Thus, the results are not statistically representative of the area and any conclusive analysis will be misleading. Furthermore, the formations of Tsotyli, Pentalofos and Eptachori are divided into members and groups. Each one of them has different properties due to different sedimentary geological histories. However, some helpful interpretations can be drawn to drive further investigation and research of the area.

The results indicate that some of the members of the formation may indeed have potentially low porosity (~5%) and permeability (< 0,01 mD). The differences between the values obtained in this work and the ones commonly pointed out in previous works, is probably related to some degree of intraformational variation of the properties and differences related to the measuring methods used. Both properties will be even lower in higher depth due to higher stress occurring, increasing the surface contact between grains. At the same time, the rock mass will be unaffected by chemical and physical weathering. As such, certain members of the Pentalofos and Eptachori formations can provide caprock layers above and below the actual reservoir member/bed. The Tsotyli formation will also provide a secure non-leaking rock mass ideal for trapping CO _2_. As such, the results pose the possibility that the area has ideal confinement layers for CO _2_ storage. Permeable zones favourable to CO _2_ storage have yet to be identified in the formation considered.

Poisson’s ratio, Young’s modulus and Brittleness Index are used in the oil/gas industry by reservoir engineers for Well Fracability as well as in injectivity of CO _2_ in saline aquifers and depleted oil/gas fields. In view of the petrophysics results, the geomechanical data should be seen as an upper boundary condition on the transboundary (contact) zone between the reservoir host rock and the cap layer rocks. 

Results for the Youngs modulus derived from P-wave propagation speed (Dynamic Elasticity modulus) and the Point load test are in relatively close agreement except for the Tsotyli formation. The latter disagreement could be the result of particular samples or the result of inelastic effects ^63^. However, it should be noted that Dynamic Elasticity modulus is a measure of the stiffness of the rock mass when it is subjected to dynamic (or rapidly changing loads), such as in the case of an earthquake or the case of vibrating structures or moving machinery. Elasticity modulus, on the other hand, is a measure of stiffness under static or constant loading. Therefore, it is expected that Dynamic Elasticity modulus derived from geophysical field methods will differ from laboratory-obtained results due to the actual sample size that introduces scale effects.

Establishing a good understanding of the Dynamic elasticity modulus of the cap and reservoir before and after CO _2_ injection is crucial to understand how the rock formations involved will be affected over time. The stiffness of the rock is important as it affects how easily the CO _2_ will flow through the reservoir and how difficult it will permeate in the cap rock. In general, the stiffer the rock, the more difficult for fluids to flow through them. Less stiff rocks deform more easily in response to the applied force imposed by the fluid that tries to flow within the pores. The results presented in Table 4 and Table 7 indicate the elasticity modulus for sedimentary rocks. Generally, the investigated rock samples are not as stiff as crystalline rocks, which are found to be in the range of >100 GPa ^64^.

All rock specimens were relatively weak when tested for tensile strength, with the lowest value of 0.8 Pa and higher 4.3 MPa. These values are typical for weathered mudstones and siltstones ^65^. However, the unweathered rocks will have a higher tensile strength. 

## Conclusions

Concluding the investigated rocks may be ideal as rock caps due to low porosity and permeability; the limited number of samples did not allow covering the entire textural variety of the formations, with the coarser lithologies, with the potential to constitute a reservoir, not being sampled.

Taking into consideration that the samples were collected at surface and the mechanical properties in depth will be forcibly different, the obtained results point to the need to control fluid pressure during injection, that should remain within specified limits; otherwise, the rock may easily fracture resulting in CO _2_ leakage or/and deform to allow the flow of CO _2_.

An important task of future and further work is to identify potential candidate members/beds of the Pentalofos and Eptachori formation with suitable reservoir properties for CO _2_ storage, i.e. porosity >10% and permeability > 100 mD.

## Ethics and consent

Ethical approval and consent were not required.

## Data Availability

Zenodo: Nuclear Magnetic Resonance values for the Eptachori, Pentalofos and Tsotyli formations in West Macedonia. https://doi.org/10.5281/zenodo.7777217 ^61^. This project contains the following underlying data: Eptachori_2_3.txt (nuclear magnetic resonance, or ‘NMR’ log). fig8.txt (NMR log). fig9.txt (NMR log). Pentalofos_3_2.txt (NMR log). Pentalofos_3_3.txt (NMR log). Pentalofos_PENT_3_1.txt (NMR log). tsotyli_TSO_1_3.txt (NMR log). Zenodo: Geomechanical laboratory investigation for the Eptachori, Pentalofos and Tsotyli formations in West Macedonia. https://doi.org/10.5281/zenodo.7849622 ^62^. This project contains the following underlying data: RawData.xlsx (raw data on Dynamic Elastic Modulus, Point Load and Schmidt Hammer). The following abbrevations were used for the samples notation: EP = Eptachori (samples were collected from ther Eptachori formation, West Macedonia, Greece) PE = Pentalofos (samples were collected from the Pentalofos formation, West Macedonia, Greece) TS = Tsotyli (samples were collected from the Tsotyli formation, West Macedonia, Greece) The numbers that follow the abbreviation such as EP1 are sequence samples extracted and cored from the bulk sample for laboratory investigation Zenodo: Nuclear Magnetic Resonance values for the Eptachori, Pentalofos and Tsotyli formations in West Macedonia. https://doi.org/10.5281/zenodo.7777217 ^61^. This project contains the following extended data: Eptachori_2_3.tif (depiction of NMR log with T2 cut-off). Fig8.tif (depiction of NMR log with T2 cut-off). fig9.tif (depiction of NMR log with T2 cut-off). Pentalofos_3_2.tif (depiction of NMR log with T2 cut-off). Pentalofos_3_3.tif (depiction of NMR log with T2 cut-off). Pentalofos_PENT_3_1.tif (depiction of NMR log with T2 cut-off). Tsotyli_TSO_1_3.tif (depiction of NMR log with T2 cut-off). Data are available under the terms of the Creative Commons Attribution 4.0 International license (CC-BY 4.0).
