# Supplementary material for: Progress for carbon dioxide geological storage in West Macedonia: A field and laboratory-based survey
Source: Open Res Eur. 2023 Aug 9;3:85. Originally published 2023 Jun 2. [Version 2] doi: 10.12688/openreseurope.15847.2 (PMC10445837; doi:10.12688/openreseurope.15847.2)
Supplement: Peer review history

# Peer review: Reviewer response for version 2

**DOI:** 10.21956/openreseurope.17754.r34363

**Published:** 2023-08-14

**Authors:** Phung Quoc HUY

**Affiliations:** 1 Asia Pacific Energy Research Centre, Kachidoki, Japan

I agree with the response from the authors to my comments.

Is the study design appropriate and does the work have academic merit?

Yes

Is the work clearly and accurately presented and does it cite the current literature?

Partly

If applicable, is the statistical analysis and its interpretation appropriate?

Yes

Are all the source data underlying the results available to ensure full reproducibility?

Partly

Are the conclusions drawn adequately supported by the results?

Partly

Are sufficient details of methods and analysis provided to allow replication by others?

Partly

Reviewer Expertise:

I am currently working at the Asia Pacific Energy Research Centre (APERC). I am interested in the coal market, clean coal technologies, energy policies, Carbon Capture, Utilization and Storage (CCUS), critical minerals, renewable energy certificates, and Low-carbon towns.

I confirm that I have read this submission and believe that I have an appropriate level of expertise to confirm that it is of an acceptable scientific standard.

# Peer review: Reviewer response for version 2

**DOI:** 10.21956/openreseurope.17754.r34362

**Published:** 2023-08-10

**Authors:** Kris Piessens

**Affiliations:** 1 Geological Survey of Belgium, Royal Belgian Institute of Natural Sciences, Brussels, Belgium

The authors replied in detail to the review comments of version 1, and to a large extend agreed to improve the publication accordingly.

Is the study design appropriate and does the work have academic merit?

Yes

Is the work clearly and accurately presented and does it cite the current literature?

Yes

If applicable, is the statistical analysis and its interpretation appropriate?

Not applicable

Are all the source data underlying the results available to ensure full reproducibility?

Yes

Are the conclusions drawn adequately supported by the results?

Yes

Are sufficient details of methods and analysis provided to allow replication by others?

Yes

Reviewer Expertise:

I am certainly less experienced with geomechanical testing, so could evaluate this only from a more generic perspective.

I confirm that I have read this submission and believe that I have an appropriate level of expertise to confirm that it is of an acceptable scientific standard.

# Peer review: Reviewer response for version 2

**DOI:** 10.21956/openreseurope.17754.r34364

**Published:** 2023-08-10

**Authors:** Rodrigo Sebastian Iglesias

**Affiliations:** 1 PUCRS, Institute of Petroleum and Natural Resources, Pontifical Catholic University of Rio Grande do Sul, Porto Alegre, State of Rio Grande do Sul, Brazil

No further comments

Is the study design appropriate and does the work have academic merit?

Yes

Is the work clearly and accurately presented and does it cite the current literature?

Yes

If applicable, is the statistical analysis and its interpretation appropriate?

Not applicable

Are all the source data underlying the results available to ensure full reproducibility?

Yes

Are the conclusions drawn adequately supported by the results?

Yes

Are sufficient details of methods and analysis provided to allow replication by others?

Yes

Reviewer Expertise:

CO2 geological storage, geochemistry, petrophysics

I confirm that I have read this submission and believe that I have an appropriate level of expertise to confirm that it is of an acceptable scientific standard.

# Peer review: Reviewer response for version 1

**DOI:** 10.21956/openreseurope.17118.r33158

**Published:** 2023-07-11

**Authors:** Phung Quoc HUY

**Affiliations:** 1 Asia Pacific Energy Research Centre, Kachidoki, Japan

Reviewer found some issues that need to be improved as follows:

1. Terminology: Several terminologies used in the article are not consistent. For example: “rock caps” in the first part of page 2 should be “cap rock”.
2. Unaxial strength results in the abstract session differ in units with the numbers in Table 2. Mpa or Gpa?
3. Need to update the current figure of the CO _2_ capture capacity in the second paragraph of page 3. Now the CO _2_ capture capacity is 46 Mtpa instead of 37 Mtpa as in the article.
4. Some parameters in the equations do not have units. For example: in equation 1, page 7.
5. In the “Methods” session, it needs to provide the diagram of the experimental apparatus. Also, the text in some paragraphs in this session seems to be in verbal English. Need to convert to academic English.
6. Several website links are in the text (page 7). It should be removed and put them in the references session.
7. Some tables should be combined into one table. For example: table 8, 9, and 10 can be combined into one table.
8. Is there any correlation in porosity value between table 8 and Figure 5? Similar questions with table 9 and figure 6; table 10 and figure 7; table 11 and figure 8; table 12 and figure 9.
9. Many references are listed in the References session, but I can not see the citation in the text. Please ensure that the reference you listed in the reference session should be cited in the text.

Is the study design appropriate and does the work have academic merit?

Yes

Is the work clearly and accurately presented and does it cite the current literature?

Partly

If applicable, is the statistical analysis and its interpretation appropriate?

Yes

Are all the source data underlying the results available to ensure full reproducibility?

Partly

Are the conclusions drawn adequately supported by the results?

Partly

Are sufficient details of methods and analysis provided to allow replication by others?

Partly

Reviewer Expertise:

I am currently working at the Asia Pacific Energy Research Centre (APERC). I am interested in the coal market, clean coal technologies, energy policies, Carbon Capture, Utilization and Storage (CCUS), critical minerals, renewable energy certificates, and Low-carbon towns.

I confirm that I have read this submission and believe that I have an appropriate level of expertise to confirm that it is of an acceptable scientific standard, however I have significant reservations, as outlined above.

# Response

**Published:** 2023-07-26

**Authors:** Pavlos Tyrologou

**Affiliations:** CERTH, Greece

Terminology: Several terminologies used in the article are not consistent. For example: “rock caps” in the first part of page 2 should be “cap rock”.

**Response: **The authors acknowledge the reviewer comment, and change was made.

 Unaxial strength results in the abstract session differ in units with the numbers in Table 2. Mpa or Gpa?

**Response: **Units are the same (Pascals), what changes is the scale. UCS obtained via point load test is presented in GPa. The units are reported in the SI system.

 Need to update the current figure of the CO2 capture capacity in the second paragraph of page 3. Now the CO2 capture capacity is 46 Mtpa instead of 37 Mtpa as in the article.

**Response:  **The authors agree with the reviewer and revised the figure to the latest one (https://www.gasworld.com/story/carbon-capture-and-storage-capacity-rises-to-244-mtpa/)

 Some parameters in the equations do not have units. For example: in equation 1, page 7.

**Response: **The authors acknowledge the reviewer comment, and changes were made.

 In the “Methods” session, it needs to provide the diagram of the experimental apparatus. Also, the text in some paragraphs in this session seems to be in verbal English. Need to convert to academic English.

**Response: **The authors see no obvious problem with the text and choose to leave the manuscript as it is. Regarding the diagrams of the apparatus, several references exist and the authors choose to economise on space.

 Several website links are in the text (page 7). It should be removed and put them in the references session.

**Response: ** The authors acknowledge the reviewer comment. This is related with the style used by the Open Research Europe.

 Some tables should be combined into one table. For example: table 8, 9, and 10 can be combined into one table.

**Response: ** The authors see no problem with the tables and choose to leave them as they are.

 Is there any correlation in porosity value between table 8 and Figure 5? Similar questions with table 9 and figure 6; table 10 and figure 7; table 11 and figure 8; table 12 and figure 9.

**Response: **The porosity value in table 8 is the direct result from the Figure 5 as it is explained the materials and methods section. For the sake of clarity the sentence was rephrased as follows Table 8 tabulates  the results from Figure 5 and both present the petrophysical results for the Tsotyli Formation. The same was done for all the other tables and figures.

 Many references are listed in the References session, but I can not see the citation in the text. Please ensure that the reference you listed in the reference session should be cited in the text.

**Response: ** The authors acknowledge the reviewer comment, and changes where needed were made.

# Peer review: Reviewer response for version 1

**DOI:** 10.21956/openreseurope.17118.r32529

**Published:** 2023-07-04

**Authors:** Kris Piessens

**Affiliations:** 1 Geological Survey of Belgium, Royal Belgian Institute of Natural Sciences, Brussels, Belgium

**General appreciation:**

 This study presents new data, be it on a very limited number of samples, which makes drawing conclusions towards the application discussed (CO _2_ geological storage) difficult. The results certainly merit being indexed.

 The main weakness of the paper, is that it fails to place the samples in a more detailed lithostratigraphic context, although this is established for this area. It is unclear why this would not be possible, and has three consequences: (1) this looks like careless sampling/field work, (2) the value of the results is diminished because of the known variation of properties at member level, and (3) drawing conclusions is strongly limited. I would strongly advice to still try and add this layer of missing information. The conclusion is weak, inaccurate and reads as a quickly added section. The authors need to discuss which conclusions they would draw.

**Grammar and spelling:**

 The manuscript contains many typos and other careless writing, and should be improved.

*Throughout text:*

- CO _2_ several times written without subscript.

*Abstract:*

- Rock caps - cap rock.

*Plain language summary:*

- Even in plain language, certain phrases need to be avoided:
  - ‘without having to fear any gas escape’ - talk about risk, not fear.
  - ‘investigate a potential country area’ - delete country?
  - ‘These samples were sent to Portugal and France’ - irrelevant information, delete.

*Introduction:*

- ‘to sequest’ - 'to sequester' (or in Europe more typical: to store).
- ‘a tonne of carbon dioxide tant range from’ - no range is given, so rephrase.
- ‘The Horizon2020’ - 'The Horizon 2020'.
- ‘successor of the StrategyCCUS project , also...’ should be 'successor of the StrategyCCUS project, also...'
- ‘CO _2_ captured from clusters of industry.’ - rephrase to ‘industrial clusters’.
- ‘Eptachori Formation and with a storage capacity’ - delete ‘and’.

*Geological setting:*

- ‘It is an elongated basin of NNW-SSE development...’ - rephrase, e.g.: 'This basin developed along a NNW-SSE elongated axis...'
- ‘alpine’ - should be 'Alpine'.

*Methods – Sampling campaign:*

- ‘strata, which could serve as CO _2_ host layers,’ - replace ‘host layers’ by either reservoir or storage complex.

*Field samples description:*

- ‘Stratigraphically from top to down, ‘ should be 'from young to old'.
- ‘CONGLOMERATE’ (and similar for other rock types) - I see no reason to capitalise?
- ‘unique sample identifiers provided by the.’ - sudden/incorrect termination of sentence.
- ‘ Laboratory of Mechanical Tests(LEM)’ should be 'Laboratory of Mechanical Tests (LEM).
- ‘KHz’ and ‘KN’ Under SI, prefix kilo = k
- ‘Samples of a square base with 5 cm edge were used, in the absence of cylindrical samples prisms of 10 cm length.’ - This sentence is quirky, but also: since you talk (correctly) about a square base, it should be 10 cm height (not length).
- ‘Five (5nr) cylindrical samples with diameter = 40 mm’ - ‘(5nr)’: simply delete? And further do not use symbols (=) in written sentences (replace by e.g. equal to)
- ‘can be obtained from NMR is the Clay-bound- water (CBW),’ should be, 'can be obtained from NMR is the Clay-bound-water (CBW),'
- ‘The test was done in seven prims with’ - should be 'prisms'.
- ‘All permeabilities were too low to be measured in the device used.’ - should be 'with the device used'.
- ‘Since and the three samples come from the same batch,’ - delete ‘and’.
- ‘from the bulk sample and extracted for petrpophysical investigation.’ - should be, 'petrophysical'.
- ‘and the Point load test are in relatively close agreement apart from the Tsotyli formation.’ - ‘apart from’ should be ‘except for’?

**Content:**

*Introduction:*

- ‘to removing eight million cars from the road each year.’ - incorrect, you remove only once, so ‘each year’ should be deleted.
- ‘scientific literature defined in Tiers1 in the’ - it is unclear what Tiers1 means/refers to?

*Methods – Sampling campaign:*

- ‘provide estimated porosity values between 7 and 25%.’ - These numbers are out of place in this section, but more importantly, you fail to discuss them later on and compare them to your own data. The difference needs to be revisited, preferably in the discussion.
- ‘some very fine-grained and thus of no or extremely low porosity, rendering them impermeable.’ - don’t mix permeability and porosity, and if you do, do this with reason.
- Fig. 3 caption: irrelevant to write that samples…were sent to, you can write that they were analysed in/by.
- ‘The results presented in Tables 7.6 and 7.8 indicate’ - Unclear which tables are references.

*Conclusion:*

- Conclusion needs to be completely revised.
- ‘Concluding the investigated rocks may be ideal as rock caps due to low porosity and permeability,’ - This is not what you argue in the discussion, there you suggest that these formations can act both as reservoir and cap rock.
- ‘but fluid pressure within the rock should remain within specified limits; otherwise, the rock may easily fracture and result in CO _2_ leakage or/and deform to allow the flow of CO _2_.’ - This sentence can not be part of a conclusion, because it is always true.

Is the study design appropriate and does the work have academic merit?

Yes

Is the work clearly and accurately presented and does it cite the current literature?

Yes

If applicable, is the statistical analysis and its interpretation appropriate?

Not applicable

Are all the source data underlying the results available to ensure full reproducibility?

Yes

Are the conclusions drawn adequately supported by the results?

Yes

Are sufficient details of methods and analysis provided to allow replication by others?

Yes

Reviewer Expertise:

I am certainly less experienced with geomechanical testing, so could evaluate this only from a more generic perspective.

I confirm that I have read this submission and believe that I have an appropriate level of expertise to confirm that it is of an acceptable scientific standard, however I have significant reservations, as outlined above.

# Response

**Published:** 2023-07-26

**Authors:** Pavlos Tyrologou

**Affiliations:** CERTH, Greece

This study presents new data, be it on a very limited number of samples, which makes drawing conclusions towards the application discussed (CO2 geological storage) difficult. The results certainly merit being indexed. The main weakness of the paper, is that it fails to place the samples in a more detailed lithostratigraphic context, although this is established for this area. It is unclear why this would not be possible, and has three consequences: 

 (1) this looks like careless sampling/field work, 

 (2) the value of the results is diminished because of the known variation of properties at member level, and 

 (3) drawing conclusions is strongly limited. I would strongly advice to still try and add this layer of missing information. The conclusion is weak, inaccurate and reads as a quickly added section. The authors need to discuss which conclusions they would draw.

**Response:** The samples are from well representative outcrops where average lithological characteristics of the formations are observed, and this is due to the exploratory character of the work, as stated in the manuscript.

 Grammar and spelling:

 The manuscript contains many typos and other careless writing, and should be improved.

**Response:** The authors acknowledge the reviewer comment, and the manuscript was checked to remove the typos

 Throughout text:

-  CO2 several times written without subscript.
-  The authors acknowledge the reviewer comment, and changes were made.

Abstract:

- Rock caps - cap rock.

**Response:**  The authors acknowledge the reviewer comment, and change was made.

 Plain language summary:

- Even in plain language, certain phrases need to be avoided:
  - ‘without having to fear any gas escape’ - talk about risk, not fear.
**Response:** The sentence was rephrased and now states: “The captured CO 2 will be stored forever, very deep into the ground, without risk of gas escaping to the atmosphere.”
  -    ‘investigate a potential country area’ - delete country?
**Response:** The change was introduced in the sentence that now states: “A team of researchers investigated a potential area close to Grevena and collected rock samples.”
  -    ‘These samples were sent to Portugal and France’ - irrelevant information, delete.
**Response:** The sentence was deleted.

Introduction:

- ‘to sequest’ - 'to sequester' (or in Europe more typical: to store).
- The change was made in the manuscript; the authors opted by “to store”
- 'a tonne of carbon dioxide tant range from’ - no range is given, so rephrase.
- The sentence was completed and now states: The positive value applications of CO 2 can also offset the cost of CCS technologies to store a tonne of carbon dioxide that range from $60 to $90 or €35 to €60 per tonne
- ‘The Horizon2020’ - 'The Horizon 2020'.
**Response:** The change was made in the manuscript.
- ‘successor of the StrategyCCUS project , also...’ should be 'successor of the StrategyCCUS project, also...'
**Response:** The change was made in the manuscript
- ‘CO2 captured from clusters of industry.’ - rephrase to ‘industrial clusters’.
  Response: The change was made in the manuscript
- ‘Eptachori Formation and with a storage capacity’ - delete ‘and’.
**Response:** The change was made in the manuscript Geological setting.
- ‘It is an elongated basin of NNW-SSE development...’ - rephrase, e.g.: 'This basin developed along a NNW-SSE elongated axis...'
**Response:** The change was made in the manuscript
- ‘alpine’ - should be 'Alpine'.
**Response:** The change was made in the manuscript

Methods – Sampling campaign:

 ‘strata, which could serve as CO2 host layers,’ - replace ‘host layers’ by either reservoir or storage complex.

**Response: **The change was made in the manuscript. The authors preferred the designation “reservoir”

 Field samples description:

 ‘Stratigraphically from top to down, ‘ should be 'from young to old'.

**Response:**

 The phrase was rewritten and currently states “Stratigraphically from the younger to the old…” 

 ‘CONGLOMERATE’ (and similar for other rock types) - I see no reason to capitalise?

**Response:** The lithological designations are following the ISO 14689: Identification and classification of rock

 ‘unique sample identifiers provided by the.’ - sudden/incorrect termination of sentence. **Response: **The phrase was rewritten and “…provided by the” was removed

 ‘Laboratory of Mechanical Tests(LEM)’ should be 'Laboratory of Mechanical Tests (LEM).

**Response:**  The correction was done

 ‘KHz’ and ‘KN’ Under SI, prefix kilo = k

**Response: **The correction was done

 ‘Samples of a square base with 5 cm edge were used, in the absence of cylindrical samples prisms of 10 cm length.’ - This sentence is quirky, but also: since you talk (correctly) about a square base, it should be 10 cm height (not length).

**Response:** The correction was made and the sentence was rewritten for clarity sake. It now states: “Prismatic samples of a square base with 5 cm edge and 10 cm height were used (due to the impossibility to produce cylindrical samples).”

 'Five (5nr) cylindrical samples with diameter = 40 mm’ - ‘(5nr)’: simply delete? And further do not use symbols (=) in written sentences (replace by e.g. equal to)

**Response:** The change was made

 ‘can be obtained from NMR is the Clay-bound- water (CBW),’ should be, 'can be obtained from NMR is the Clay-bound-water (CBW),'

**Response:** The authors see no problem with the phrase and choose to leave it as it is. 

 ‘The test was done in seven prims with’ - should be 'prisms'.

**Response: **The correction was done

 ‘All permeabilities were too low to be measured in the device used.’ - should be 'with the device used'.

**Response:** The correction was done

 ‘Since and the three samples come from the same batch,’ - delete ‘and’.

**Response: **The correction was done

 ‘from the bulk sample and extracted for petrpophysical investigation.’ - should be, 'petrophysical'.

**Response:** The correction was done

 ‘and the Point load test are in relatively close agreement apart from the Tsotyli formation.’ - ‘apart from’ should be ‘except for’?

**Response: **The change was made

 Content: Introduction:

 ‘to removing eight million cars from the road each year.’ - incorrect, you remove only once, so ‘each year’ should be deleted.

**Response:**

 The change was made

 ‘scientific literature defined in Tiers1 in the’ - it is unclear what Tiers1 means/refers to?

**Response:**

 This is an established designation for the maturity level of the information. For the sake of clarity the phrase was rewritten as “Previously, in STRATEGY CCUS a conservative geological modelling approach based on existing scientific literature attributed the Tiers1classification for the Mesohellenic Trough, which contains Pentalofos Formation with a CO 2 capacity up to 1 Gt and Eptachori Formation with a storage capacity up to 0.85 Gt of CO 2 “

 Methods – Sampling campaign:

 ‘provide estimated porosity values between 7 and 25%.’ - These numbers are out of place in this section, but more importantly, you fail to discuss them later on and compare them to your own data. The difference needs to be revisited, preferably in the discussion.

**Response:**

 The authors partially agree with the reviewer, on the need to revisited the divergence between the data from previous works with the data from the current work in the discussion, although they do not find the information in the Methods-Sampling Campaign completely out of place. At the discussion the following sentence was added to justify the apparent differences between old data and new data: “The differences between the values obtained in this work and the ones commonly pointed out in previous works, is probably related to some degree of intraformational variation of the properties and differences related to the measuring methods used.”

 ‘some very fine-grained and thus of no or extremely low porosity, rendering them impermeable.’ - don’t mix permeability and porosity, and if you do, do this with reason.

**Response:** The authors understand that porosity and permeability are different properties. However, considering that the are no information on the permeability of the formations an empirical approached allow us to carefully state that very-fine sandstones are more likely to be impermeable, based on the work of other authors devoted to the permeability-porosity relation (Pape et al., 2000 and Kameda et al. 2006). For the sake of clarity the sentence was rephrased as follows: ”For the western areas not covered by the Tsotyli strata, the higher layers of Pentalofos and Eptachori Formations could potentially serve as cap-rock themselves because they consist of alternating layers with alternating different characteristics, some very fine-grained and thus of no or extremely low porosity, which predicts conditions of very low permeability”

 Fig. 3 caption: irrelevant to write that samples…were sent to, you can write that they were analysed in/by.

**Response:** The caption was changed and now states: “Figure 3. Bulk samples collected during the walk over survey and sent toanalysed in France:  by French Institute of Petroleum (IFP) Energies nouvelles – Earth Sciences and Environmental Technologies and in Portugal:  by the Departamento de Geociências Universidade de Évora for petrophysical and geomechanically laboratory investigation respectively.”

 ‘The results presented in Tables 7.6 and 7.8 indicate’ - Unclear which tables are references.

**Response:**

 The correction was made, and now refers to Tables 4 and 7

 Conclusion:

 Conclusion needs to be completely revised.

 ‘Concluding the investigated rocks may be ideal as rock caps due to low porosity and permeability,’ - This is not what you argue in the discussion, there you suggest that these formations can act both as reservoir and cap rock.

 ‘but fluid pressure within the rock should remain within specified limits; otherwise, the rock may easily fracture and result in CO2 leakage or/and deform to allow the flow of CO2.’ - This sentence can not be part of a conclusion, because it is always true.

**Response:** The Conclusions were rewritten as follows: “Concluding the investigated rocks may be ideal as rock caps due to low porosity and permeability; the limited number of samples did not allow covering the entire textural variety of the formations, with the coarser lithologies, with the potential to constitute a reservoir, not being sampled. 

 Taking into consideration that the samples were collected at surface and the mechanical properties in depth will be forcibly different, the obtained results point to the need to control fluid pressure during injection, that should remain within specified limits; otherwise, the rock may easily fracture resulting in CO 2 leakage or/and deform to allow the flow of CO 2. 

 An important task of future and further work is to identify potential candidate members/beds of the Pentalofos and Eptachori formation with suitable reservoir properties for CO 2 storage, i.e. porosity >10% and permeability > 100 mD.”

# Peer review: Reviewer response for version 1

**DOI:** 10.21956/openreseurope.17118.r32523

**Published:** 2023-06-26

**Authors:** Rodrigo Sebastian Iglesias

**Affiliations:** 1 PUCRS, Institute of Petroleum and Natural Resources, Pontifical Catholic University of Rio Grande do Sul, Porto Alegre, State of Rio Grande do Sul, Brazil

The authors present a simple, well-constrained study of the potential capability of a West-Macedonian (Mesohellenic) sedimentary basin for CO _2_ geological storage, using geo-mechanical and petrophysical characterization methods.

 The manuscript is overall well-written, clear and objective, apart from a few typos and minor inconsistencies (currency formats and project titles). The methods employed are adequate, although the results and discussions are rather limited due to small number and type of samples (outcrops), and lack of replicate analyses.

 Nevertheless, the authors made these limitations very clear, with fitting conclusions, laying out a good foundation for further studies in the area.

Is the study design appropriate and does the work have academic merit?

Yes

Is the work clearly and accurately presented and does it cite the current literature?

Yes

If applicable, is the statistical analysis and its interpretation appropriate?

Not applicable

Are all the source data underlying the results available to ensure full reproducibility?

Yes

Are the conclusions drawn adequately supported by the results?

Yes

Are sufficient details of methods and analysis provided to allow replication by others?

Yes

Reviewer Expertise:

CO2 geological storage, geochemistry, petrophysics

I confirm that I have read this submission and believe that I have an appropriate level of expertise to confirm that it is of an acceptable scientific standard.
